# Effects of Embedded TiO_2−x_ Nanoparticles on Triboelectric Nanogenerator Performance

**DOI:** 10.3390/mi9080407

**Published:** 2018-08-17

**Authors:** Hyun-Woo Park, Nghia Dinh Huynh, Wook Kim, Hee Jae Hwang, Hyunmin Hong, KyuHyeon Choi, Aeran Song, Kwun-Bum Chung, Dukhyun Choi

**Affiliations:** 1 Department of Mechanical Engineering, Kyung Hee University, 1732 Deogyeong-daero, Giheung-gu, Yongin-Si, Gyeonggi-do 446-701, Korea; mnphwcj@gmail.com (H.-W.P.); hdnghia567@gmail.com (N.D.H.); choice124@hanmail.net (W.K.); chgw8584@hanmail.net (H.J.H.); 2 Department of Physics and Semiconductor Science, Dongguk University, Seoul 100-715, Korea; ggm0218@naver.com (H.H.); cransia@hanmail.net (K.C.); aeransong@dongguk.edu (A.S.)

**Keywords:** TiO_2−x_ nanoparticle, high dielectric constant, triboelectric nanogenerators, oxygen vacancy

## Abstract

Triboelectric nanogenerators (TENGs) are used as self-power sources for various types of devices by converting external waves, wind, or other mechanical energies into electric power. However, obtaining a high-output performance is still of major concern for many applications. In this study, to enhance the output performance of polydimethylsiloxane (PDMS)-based TENGs, highly dielectric TiO_2−x_ nanoparticles (NPs) were embedded as a function of weight ratio. TiO_2−x_ NPs embedded in PDMS at 5% showed the highest output voltage and current. The improved output performance at 5% is strongly related to the change of oxygen vacancies on the PDMS surface, as well as the increased dielectric constant. Specifically, oxygen vacancies in the oxide nanoparticles are electrically positive charges, which is an important factor that can contribute to the exchange and trapping of electrons when driving a TENG. However, in TiO_2−x_ NPs containing over 5%, the output performance was significantly degraded because of the increased leakage characteristics of the PDMS layer due to TiO_2−x_ NPs aggregation, which formed an electron path.

## 1. Introduction

Triboelectric nanogenerators (TENGs) have demonstrated the ability to convert surrounding ambient mechanical energy into electricity based on the coupling effects of contact triboelectrification and electrostatic induction. However, the need for a low-cost method and an improvement in output performance remain a challenge in TENG fabrication. A practical application to harvest mechanical energy is also required. In general, the two key factors that significantly affect the output performance of TENGs are the tribo-material and the effective contact area of the friction layers. Another study showed that the surface charge density enhanced the triboelectric material and that the surface morphologies in micro/nanopatterning control the TENGs performance [1,2]. As shown in 2012 by Wang’s group [3], TENGs operate in four basic modes: vertical contact-separation mode; lateral sliding mode; single electrode mode, and freestanding triboelectric-layer mode. Among these working modes, the vertical contact–separation mode was introduced first and has become the primary mode due to its simple operation, durability, stable and high-output performance. Basically, using the electrostatic charges created on the surfaces of two dissimilar materials when they are brought into contact, the tribo-charges can generate a potential drop when two surfaces are separated by an external force and, thus, drive electron flow between two electrodes connected on both sides of the tribo-material.

Recently, TENG development has become very common in many fields, such as energy harvesting, self-powered sensors, self-charging systems, or self-powered wearable electronic devices. TENGs has been investigated to obtain higher electricity output using surface charge density enhancement via ionized injection, which alters the effective contact area between the friction tribo-layers. This improvement is through its surface patterning or through new materials developed using functional group attachment. However, the simple, low-cost, and flexible structure of TENGs through embedded nanoparticles (NPs) is still being considered as a way to increase the capacitance of TENGs. By modifying the inside polymers through filling with nanoparticles and forming pores, many studies have reported the advantages of an embedded high dielectric constant material in polydimethylsiloxane (PDMS), such as BaTiO_3_, SrTiO_3_ [4,5], Au, or Ag nanoparticles [6], for improving TENG capacitance. In addition, the output performance of TENG can be increased by adjusting the distribution depth of the tribo-charges in the friction layer as described by Cui et al. [7]. C. Wu et al. [8] also demonstrated that the charge density in the friction layer is enhanced by utilizing a reduced graphene oxide (rGO) acting as electron-trapping sites [8]. These methods can lead to the higher output performance of TENGs, but are still limited in real applications due to the complexity, high-cost, and lack of both physical characteristic and analysis properties for understanding embedded NP behaviors inside the tribo-polymers layer. Moreover, oxygen vacancies in the oxide nanoparticles are electrically positive charges [9], which is an important factor that can contribute to the exchange and trapping of electrons when driving a TENG. In previous studies, however, the effect of oxygen vacancies within the tribo-material surface according to the oxide nanoparticle embedded ratio was not studied.

In this work, we report a significant improvement of TENG performance by considering TiO_2−x_ NPs embedded in PDMS as a function of weight ratio. Through this work, the optimal TiO_2−x_ NPs embedded PDMS is determined, and we investigated the correlation between TENG output behavior and the physical properties of the TiO_2−x_ NPs-embedded PDMS, such as the surface potential, dielectric properties, oxygen vacancy, and electronic structures. Different TiO_2−x_ NPs embedded according to PDMS weight ratios from 0 to 30% were chosen for tribo-layer fabrication. The results show a significantly enhanced TENG output upon the use of TiO_2−x_ NPs embedded PDMS as compared to a pristine PDMS tribo-layer. The optimal TiO_2−x_ NPs embedded PDMS with a 5% weight ratio exhibited the highest voltage of 180 V and current of 8.15 µA under a 5 N pushing force and 5 Hz pushing frequency. Furthermore, for practical applications, a portable windmill system integrated with our optimal TENGs using a low-cost, simple fabrication process involving plastic bottle waste is proposed.

## 2. Materials and Methods

### 2.1. The Fabrication Process of the PDMS Layer and TiO_2−x_ NPs Embedded PDMS Layer

Two hundred micrometers thick PDMS and TiO_2−x_ NPs embedded PDMS layers were fabricated by simple imprint lithography (SIL) using a doctor–blade and a Teflon template as a mold. The Teflon template was cleaned with acetone, ethanol, and deionized (DI) water, consecutively, and then gently dried with dry nitrogen (N_2_) gas. To prevent the PDMS layer from sticking to the mold, we treated the surface of the Teflon mold with heptadecafluoro-1, 1, 2, 2-tetrahydrodecyltrichlorosilane (HDFS) to make its surface hydrophobic. The PDMS was prepared as a mixture of base resin and curing agent (Sylgard 184 A: Sylgard 184 B, Dow Corning Co., Midland, MI, USA) with a weight ratio of 10:1. After a degassing process under vacuum for approximately 30 min, the PDMS mixture was then laminated to the Teflon mold by a doctor–blade technique and cured at 80 °C for six hours. The 200 µm thick PDMS layer was then carefully peeled off the Teflon mold. The TiO_2−x_ NPs-embedded PDMS layer was fabricated similarly, except the embedding process of TiO_2−x_ NPs (US Research Nanomaterials, Inc. TiO_2−x_ Nanoparticles, rutile, high purity, 99.9+%) utilized different mass ratios from 5 to 30%, dispersed into the PDMS mixture.

### 2.2. Fabrication and Output Measurement of the TENGs

To design the vertical contact–separation mode TENG, the top electrode was prepared with a 3 × 3 cm^2^, 80 µm-thick commercial aluminum foil, which was cleaned by ethanol and dried using N_2_ gas. The aluminum foil was then attached to the polylactic acid (PLA) substrate with double-sided foam tape. Subsequently, to prepare the TENG bottom electrode, the PDMS layer and TiO_2−x_ NPs embedded PDMS layer were carefully laminated onto the aluminum plate. Figure S1 (Supporting Information) shows a schematic of the fabrication process for TiO_2−x_ NPs embedded TENG.

### 2.3. Analysis of the TiO_2−x_ NPs-Embedded TENG

The physical structure of TiO_2−x_ nanoparticles was analyzed using X-ray diffraction (XRD). The surface morphology characteristics of the PDMS layer and TiO_2−x_ NPs-embedded PDMS layer were examined using scanning electron microscopy (SEM) and atomic force microscopy (AFM). To measure the TENG output performance, a force was applied using a pushing tester. The TENG output voltage was measured using a Tektronix MDO3052 (Tektronix, Inc., Beaverton, OR, USA) mixed-domain oscilloscope with an input impedance of 40 MΩ, and output current measurements were captured using a Stanford Research Systems SR570 low-noise current preamplifier (Stanford Research System, Sunnyvale, CA, USA) connected to the Tektronix MDO3052 mixed domain oscilloscope. The TENG performance was measured at a working frequency of 5 Hz and a force of 5 N; the gap between the two electrodes was 10 mm. The chemical bonding states and composition of PDMS and TiO_2−x_ NPs-embedded PDMS layers were also examined by X-ray photoelectron spectroscopy (XPS) with a pass energy of 20 eV, using a monochromatic Al K*α* source. The change in the unoccupied state in the conduction band according to the TiO_2−x_ percentage was investigated using X-ray absorption spectroscopy (XAS). XAS experiments were performed at the 10 D beamline in the Pohang Accelerator Laboratory (PAL), Korea. In addition, the triboelectric charges of PDMS, according to the TiO_2−x_ embedded weight ratio, were quantitatively studied using Kelvin probe force microscopy (KPFM, N8-NEOS, Bruker, Billerica, MA, USA) and contact potential difference (CPD) measurements.

## 3. Results and Discussion

**Figure 1:**
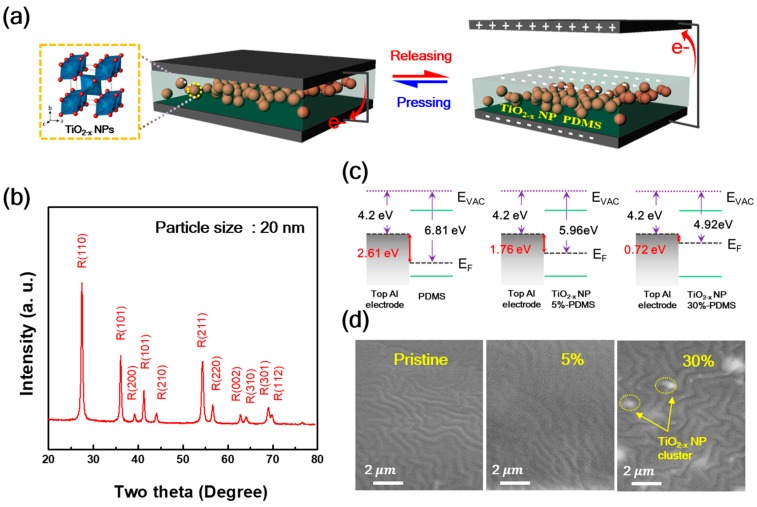
(**a**) Schematic of the vertical contact–separation mode triboelectric nanogenerators (TENG) with TiO_2−x_ NPs embedded in polydimethylsiloxane (PDMS) as friction layers; alternating current is generated upon pressing and releasing; (**b**) X-ray diffraction (XRD) spectra of the TiO_2−x_ nanoparticles (NPs); (**c**) The energy band diagram, and (**d**) Scanning electron microscope (SEM) image of PDMS as a function of the TiO_2−x_ NPs embedded weight ratio.

The proposed TENG was operated using a simple model of contact–separation, as depicted in Figure 1a. The TiO_2−x_ NPs-embedded PDMS layer was laminated on the Al electrode as a negative tribo-material. When the TiO_2−x_ NPs-embedded PDMS layer comes into contact with the top layer Al electrode (i.e., positive tribo-material) as well, the TiO_2−x_ NPs-embedded PDMS layer becomes negatively charged while the Al electrode is positively charged because of the different electron affinities. During the release process, potential is created between the two opposite electrostatic charges. The potential across these electrodes leads to an electron flow through the external circuit from the bottom Al electrode to the top to obtain electrical equilibrium. After full separation, the electrodes revert back to their original states, and at last, the tribo-charge distribution reaches electrical equilibrium. Applying a continuous external pushing force to the TENGs, repeated TENG operation can be observed. Since the titanium oxide materials have various physical structures, such as anatase, rutile, and bookite, and their electrical, optical, and physical properties depend on their physical structure [10], XRD patterns of the TiO_2−x_ NPs were investigated, as shown in Figure 1b. The TiO_2−x_ NPs represent the typical polycrystalline rutile structure with diffraction peaks of (110), (101), (200), (101), (210), (211), (220), (002), (310), (301), and (112), and the calculated particle size was about 20 nm through main XRD peak of (110). [11,12] The energy band diagram of the PDMS layer and TiO_2−x_ NPs-embedded PDMS layer with 5% and 30% are shown in Figure 1c. As TiO_2−x_ NP increased, the work function difference between the two contact materials is reduced from 2.61 eV to 0.72 eV. This implies that the transport of electrons from the top Al electrode can be reduced.

Figure 1d shows the scanning electron microscope (SEM, compact SEM GENESIS-1000, Emcrafts, Korea) images of the PDMS layer and TiO_2−x_ NPs embedded PDMS layer with 5% and 30%. The surface morphology of these PDMS and TiO_2−x_ NPs-embedded PDMS layers are uniformly wrinkle-like and similar, except for the higher weight ratio TiO_2−x_ NPs-embedded PDMS layers. The average roughness of these TiO_2−x_ NPs-embedded PDMS layers is confirmed by the AFM image, and the mean surface roughness of the PDMS layer and TiO_2−x_ NPs-embedded PDMS layer with 5% and 30% was 17.8, 22.1, and 19.8 nm, respectively. (Not shown here).

**Figure 2:**
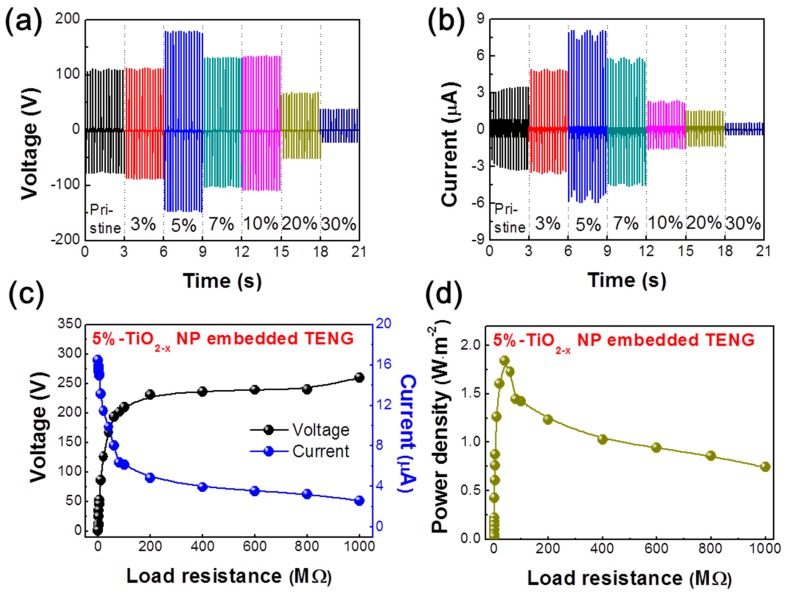
Effects of the output power of TENGs caused by embedded TiO_2−x_ NPs. (**a**) Output voltage and (**b**) current of the TiO_2−x_ NP embedded TENGs as a function of the TiO_2−x_ NPs embedded weight ratio (measured under a relative humidity of ~55%). Influence of external load resistance on the (**c**) output voltage/current and (**d**) power density of the TENG under a pushing force of 5 N and a pushing frequency of 5 Hz.

Figure 2a,b show the output voltage and current characteristics of the TENG without/with TiO_2−x_ NPs as a function of weight ratio (measured under a relative humidity of ~55% and room temperature of 27 °C). As the weight ratio of TiO_2−x_ NPs increased from 0% (pristine PDMS) to 5%, the output voltage and current increased significantly from 112 V and 3.5 µA to 180 V and 8.15 µA, respectively. However, at weight ratios greater than 5%, the output voltage and current drastically decreased to 38.7 V and 0.6 µA, respectively. Moreover, to examine the effect of the pushing force on the output performance of the TENG device with pristine PDMS, 5% (optimal weight ratio) and 30% TiO_2−x_ NPs-embedded PDMS, the output voltage and current of the TENG were measured and analyzed at different pushing forces ranging from 5 to 20 N and pushing frequency of 5 Hz (Figure S2—Supporting Information). It clearly indicates that the peak voltage and current of the TENG were increased from 48.2 V and 1.6 µA to 91.8 V and 2.9 µA for the pristine TENG, from 64.9 V and 3.8 µA to 126.8 V and 6.1 µA for 5%, and that of 30% TiO_2−x_ NPs-embedded PDMS based TENG increased from 30.6 V and 0.9 µA to 58.7 V and 2.1 µA by enhancing the applied pushing forces from 5 N to 20 N, respectively. The highest output voltage and current values of 126 V and 6.1 µA were obtained under the externally applied force of 20 N. This result is mainly attributed to the surface potential dramatically changing among these kind of TENGs. This was especially true for the 30% TiO_2−x_ NPs-embedded PDMS which became a positively charged material, leading to the drop of the TENG output performance, even though a strong applied pushing force. In addition, the trend of the TENG output among pristine, 5%, and 30% is the same as the measurement at 5 N and 5 Hz shown in Figure 2. It was noted that the surrounding conditions were 75% relative humidity and 32 °C room temperature for the different applied forces measurements. Figure 2c shows the effect of voltage and current characteristics of the TENG as a function of external load resistance ranging from 0.1 to 1000 MΩ. These measurements were performed under a pushing force and frequency of 5 N and 5 Hz, respectively. As shown in Figure 2c, the TENG voltage increased by increasing the resistance from 0.1 to 200 MΩ, while the current value followed the opposite trend. Afterward, both the voltage and current became saturated at very high load resistances (i.e., 200–1000 MΩ), and the TENG exhibited the highest voltage and lowest current values ranging from 230.6–260 V and 4.8–2.6 µA. In addition, the average power density (*P_avg_*) was calculated by the following the equation:

(1) $$P_{avg}=\frac{VI}{S}$$

where *V* and *I* are the output peak voltage and current values of the TENG at various load resistances, respectively, and *S* is the active area (i.e., 9 cm^2^) of the TENG. Figure 2d shows the effect of the TENG output power density as a function of external load resistance. Following Figure 2d, the *P_avg_* values of TENG increased by increasing the load resistance and then decreased at a relatively high load resistance. Specifically, at the moderate load resistance of 40 MΩ, the TENG exhibited a maximum power density of 1.84 W.m^−2^, under the pushing force and frequency of 5 N and 5 Hz, respectively. More discussion is provided below regarding the output performance behavior of the TENGs and the PDMS properties according to the TiO_2−x_ NP embedding ratio.

**Figure 3:**
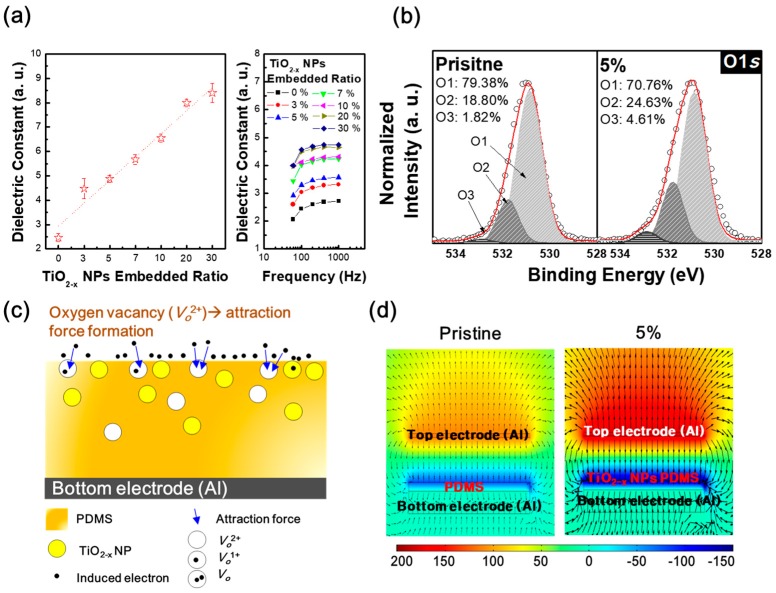
(**a**) Dielectric constant of the TiO_2−x_ NPs embedded PDMS layers according to the frequency with different TiO_2−x_ NPs weight ratios; (**b**) De-convoluted X-ray photoelectron spectroscopy (XPS) spectra of oxygen (O) 1*s* states of pristine PDMS layer and TiO_2−x_ NPs 5% embedded PDMS layer; (**c**) Schematic of the electron attraction mechanism according to oxygen vacancies of the PDMS surface, and (**d**) COMSOL simulation results of the pristine TENG and TiO_2−x_ NPs 5% embedded TENG.

Previous studies show that as the embedded weight ratio of NPs with high dielectric constants increases, the TENG output performance was improved because of the increasing dielectric constant of the tribo-material [13]. Our results show the same trends as previous studies, as shown in Figure 3a. In addition, the dielectric constant according to frequency does not change significantly. However, we provide below not only an increase in the dielectric constant of the tribo-material but also suggest an additional mechanism based on the role of oxygen vacancy in TiO_2−x_ NP embedded PDMS to effectively enhance TENG performance. To investigate changes of chemical bonding state within the PDMS layer according to the amount of embedded TiO_2−x_ NPs, the oxygen (O) 1*s* spectra of PDMS and TiO_2−x_ NPs 5% embedded PDMS layers were observed via XPS, as shown in Figure 3b. For the detailed analysis of chemical bonding states, the O 1*s* spectra were carefully normalized and de-convoluted with three different Gaussian peaks as indexed with O1, O2, and O3 centered at 530.9 eV, 531.8 eV, and 532.9 eV, respectively. Each of the representatively assigned peaks from a low binding energy is related to the oxygen state in the metal-oxide lattices, the oxygen-deficient state, and chemisorbed or dissociated oxygen states, or OH^−^ impurities, respectively [14,15]. Among them, the relative area of the oxygen-deficient peak (O2) is dramatically increased from 18.80% to 24.63% after TiO_2−x_ NPs embedding, which is remarkable. Moreover, we measured and de-convoluted O 1*s*’ peak of other films that have different concentration, as shown in Figure S3. In the result, the oxygen-deficient states were similar regardless of increases of TiO_2−x_ concentration. This saturation can be observed due to the formation of TiO_2−x_ clusters in the composite film (see Figure 1d). The TiO_2−x_ nanoparticles in a cluster act like a single particle. This means that the number of active oxygen deficient states are as same as a single TiO_2−x_ nanoparticle. Due to the oxygen vacancies being electrically positively charged, the increment of oxygen vacancies of the negative tribo-material surface could induce electron flow from the top electrode by the attraction force, as shown in Figure 3c. Therefore, the TiO_2−x_ NPs-embedded PDMS layer has a larger oxygen-deficient state by oxygen vacancies, which can contribute to an enhanced TENG performance. To theoretically understand the output performance enhancement of the TiO_2−x_ NPs-embedded TENG, a COMSOL Multiphysics (5.0, COMSOL, Inc.) simulation was conducted. Figure 3d shows the corresponding results for the triboelectric potential distributions of pristine TENGs and TiO_2−x_ NPs-embedded TENG, respectively. The details of the COMSOL Multiphysics simulation parameters and equations are provided in our previous study, Reference [16]. As a result, the COMSOL simulations showed that the 5% TiO_2−x_ NPs-embedded TENG can exhibit higher performance than the pristine TENG.

**Figure 4:**
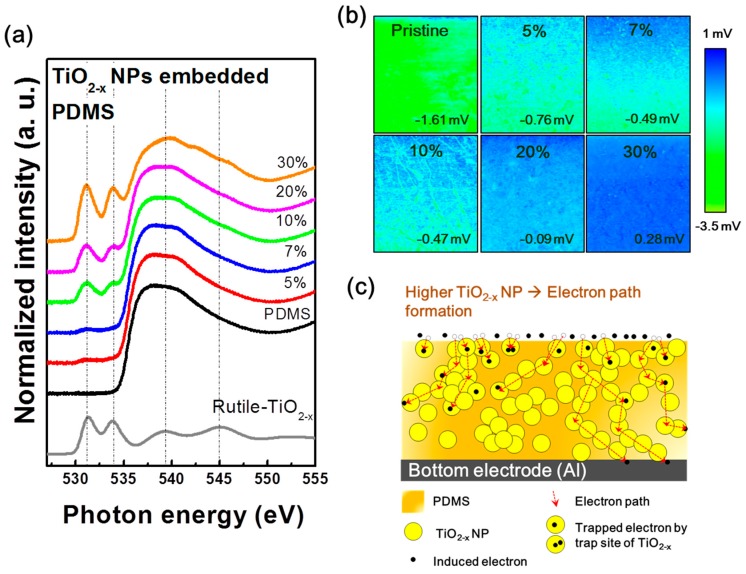
(**a**) O-K edge X-ray absorption spectroscopy (XAS) spectra and (**b**) Kelvin probe force microscopy (KPFM) images of PDMS layers as a function of the TiO_2−x_ NPs embedded weight ratios; (**c**) Schematic of the electron leakage mechanism according to electron path formation.

**Figure 5:**
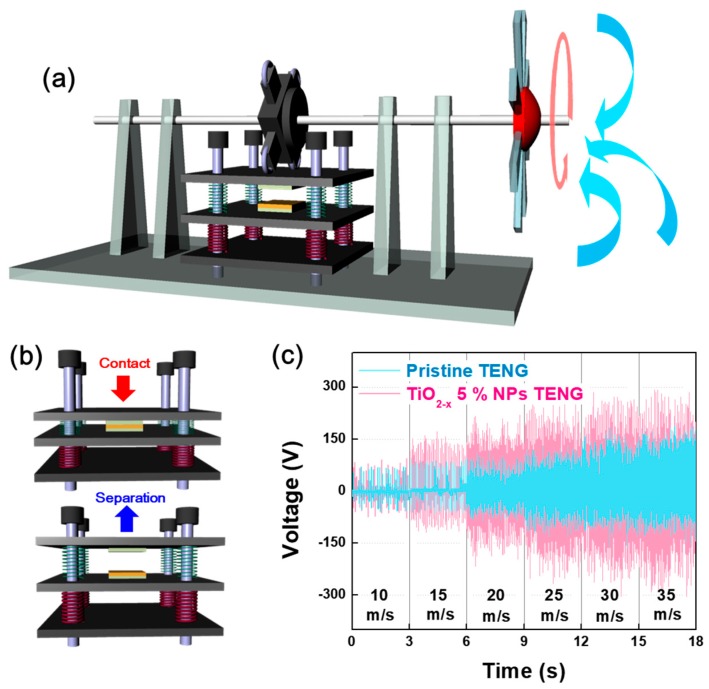
(**a**) Schematic of the windmill system and (**b**) TENG operation in the vertical direction repetitively switching between the contact and separation modes; (**c**) Measured voltage of the TiO_2−x_ NPs 0% (pristine TENG) and 5% embedded TENG at different wind speeds applied to the windmill system.

To elucidate the cause of the significantly decreased output performance of TENG containing over 5% embedding TiO_2−x_ NPs, the correlation between the electronic structures of the TiO_2−x_ NPs-embedded PDMS and the energy conversion mechanism of the TENG were investigated. Figure 4a shows the hybridized molecular orbital structures in the conduction band of the TiO_2−x_ NPs-embedded PDMS as a function of the weight ratio of TiO_2−x_ NPs measured by XAS. The normalized intensities of the oxygen K-edge spectra of pristine PDMS and TiO_2−x_ NPs-embedded PDMS layers directly reflect the unoccupied hybridized states by the transition of electrons from the occupied O 1*s* state [17]. Figure 4a co-plots the normalized O-K edge XAS spectrum of TiO_2−x_ NPs (gray lines) for easy comparison with the changes in the TiO_2−x_ NPs-embedded PDMS layer spectra. In particular, XAS analysis is a surface sensitive analytical method that measures the current flowing on the sample surfaces after incident X-ray exposure. Therefore, this is suitable for analyzing tribo-materials whose output performance changes according to surface conditions. The XAS spectra of TiO_2−x_ NPs are located in the two strong resonance peaks at 531.5 and 534 eV [18]. With an increased weight ratio of TiO_2−x_ NPs from 0 to 30%, the electronic structure of PDMS is clearly changed by the addition of the electronic structure of TiO_2−x_ NPs. These changes in the tribo-material surface can cause changes in the tribo-series, resulting in reduced tribo-electrification effects. For the experimental demonstration of our claim, KPFM measurements were carried out to detect the surface potential of different surfaces according to the weight ratio of TiO_2−x_ NPs, as shown in Figure 4b. As the weight ratio of TiO_2−x_ NPs increased from 0 to 30%, the average surface potential dramatically changed. Specifically, the TiO_2−x_ NPs 30% sample became a positively charged material. Moreover, considering the SEM image in Figure 1d, the electron path can be formed due to NPs clustering in the case of the TENG containing over 5% embedding TiO_2−x_ NPs. The electron path formed by the NPs cluster can move the electrons induced on the surface and increase the leakage current, as shown in Figure S4 (supporting information), which may be a reasonable cause to reduce TENG performance above 5% embedding TiO_2−x_ NPs, as shown in Figure 4c. To demonstrate this with a practical application, we designed a windmill system to harvest the wind energy utilizing the pristine TENG and optimal TENG devices (TiO_2−x_ NPs 5% embedded TENG), as shown in Figure 5a. In the presence of wind flow, the windmill blades are forced to rotate and lead to the rotation of a quad nose cam. Subsequently, the quad nose cam transmits its rotation motion to linear motion on the top plate of the TENG. Resulting from that motion, the TENG operates in the vertical direction via contact and separation mode repeatedly (Figure 5b), which produces the TENG electrical output. Moreover, the relation of wind speed and output performance of the windmill-integrated TENG was also analyzed at various wind speeds, as shown in Figure 5c. The measured voltage is clearly shown in the output performance of both the pristine and optimal TENGs, which show linear enhancement upon increasing the wind speed applied to the windmill blades. The peak voltage values of the windmill attached to pristine TENG at the wind speeds of ~10, 15, 20, 25, 30, and 35 are 70, 92, 114, 130, 182, and 190, and that for the windmill integrated with an optimal TENG are 94, 172, 220, 268, 304, and 304, respectively. The output data in Figure 5c also show a linear enhancement regarding the number of pushing cycles, i.e., pushing frequency and force on the top plate of the TENG as the wind speed increases. With faster wind speeds, the output performance of the windmill TENG can be enhanced. The results clearly demonstrate that our windmill TENG system can efficiently harvest wind energy.

## 4. Conclusions

In summary, we demonstrated a facile approach to improve the output performance of TENGs by embedding high dielectric TiO_2−x_ nanoparticles in the PDMS layer. The output performance of the TiO_2−x_ NPs embedded (5%) TENG showed the highest output voltage and current of 180 V and 8.15 µA, respectively, which is strongly related to the change of oxygen vacancies on the PDMS surface as well as the increased dielectric constant. Since the oxygen vacancies are positively charged, the increment of oxygen vacancies of the negative tribo-material surface could induce electrons from the top electrode by attraction force. Therefore, the TiO_2−x_ NPs-embedded PDMS layer has larger oxygen vacancies, which can contribute to the enhanced TENG performance. In contrast, with TiO_2−x_ NPs embedded samples over 5%, the output voltage and current were drastically decreased to 38.7 V and 0.6 µA, respectively. This significant degradation of output performance is due to variation of the tribo-series, resulting in reduced tribo-electrification effects. In addition, the electron path could be formed due to TiO_2−x_ NPs clustering, which can move the electrons induced on the surface thereby increasing leakage current. Last, our optimal TENG was also presented as a practical application for wind energy harvesting and could obtain 424 V of the peak output voltage via the proposed windmill.

## Supplementary Materials

The following are available online at http://www.mdpi.com/2072-666X/9/8/407/s1, Figure S1: Schematic of the fabrication process for TiO_2−x_ NPs embedded TENG, Figures S2: TENG performance according to the different applied input forces, Figures S3: Change of oxygen deficient states with various TiO_2−x_ NP concentrations, Figures S4: Leakage current characteristics of PDMS layer as a function of TiO_2−x_ NPs embedded ratio.
