# Supplementary material for: Effects of Embedded TiO2−x Nanoparticles on Triboelectric Nanogenerator Performance
Source: Micromachines (Basel). 2018 Aug 17;9(8):407. doi: 10.3390/mi9080407 (PMC6187469; doi:10.3390/mi9080407)
Supplement: Supplementary file 1 [file micromachines-09-00407-s001.pdf]

micromachines

Supplementary Materials: Effects of Embedded TiO_{2-x} Nanoparticles on Triboelectric Nanogenerator Performance

Hyun-Woo Park, Nghia Dinh Huynh, Wook Kim, Hee Jae Hwang, Hyunmin Hong, KyuHyeong Choi, Aeran Song, Kwun-Bum Chung, and Dukhyun Choi

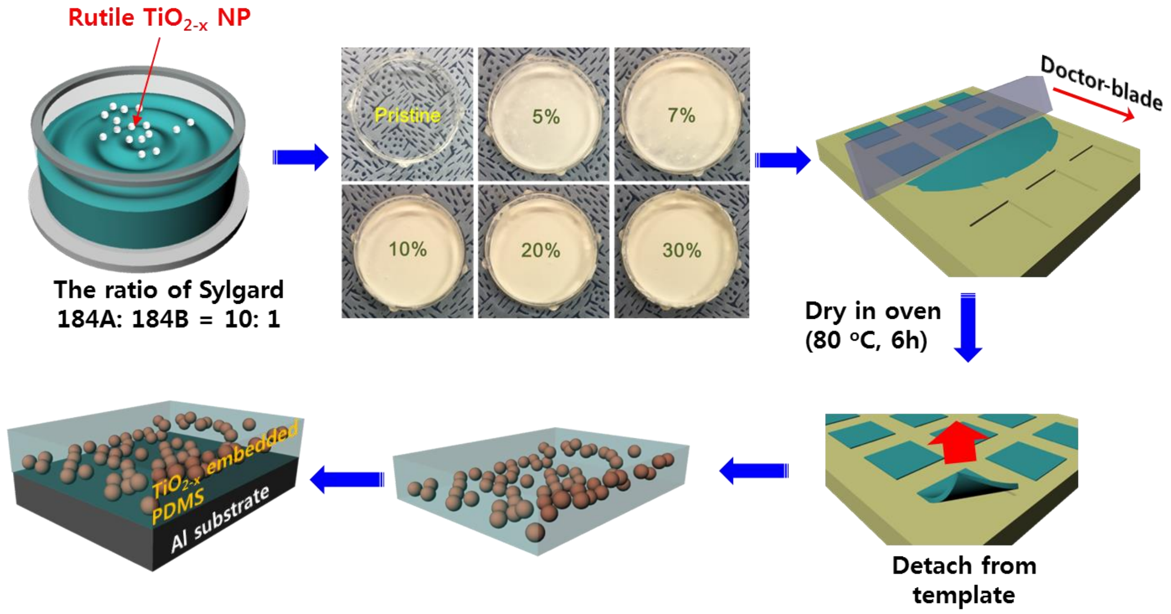

Figure S1. Schematic of the fabrication process for TiO_{2-x} NPs embedded TENG.

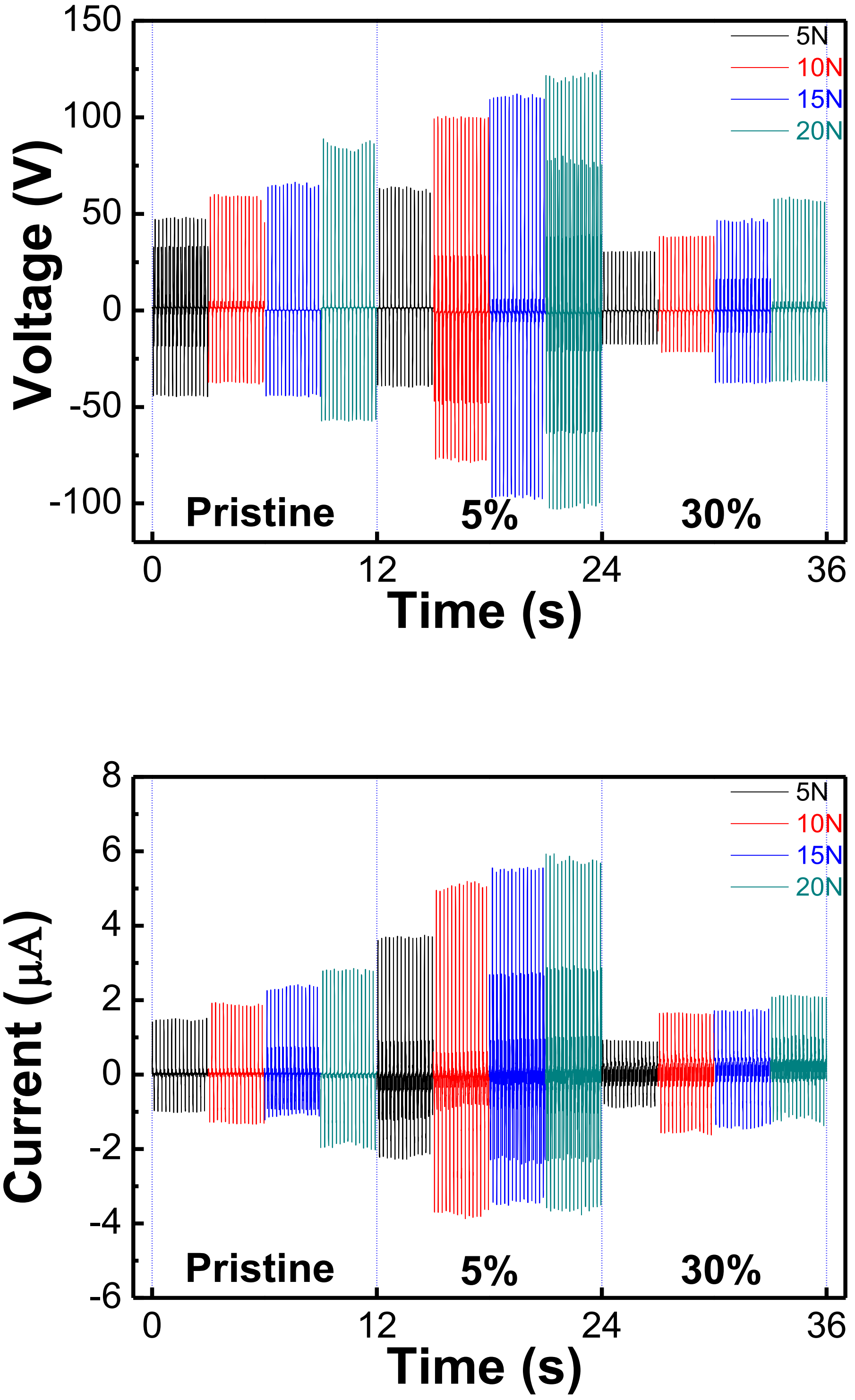

Figure S2. The voltage and current output performance of the TiO_{2-x} NPs-embedded PDMS based TENG with TiO_{2-x} NPs weight ratio of 0%, 5% and 30% at different applied forces ranging from 5 N to 20 N and pushing frequency of 5 Hz.

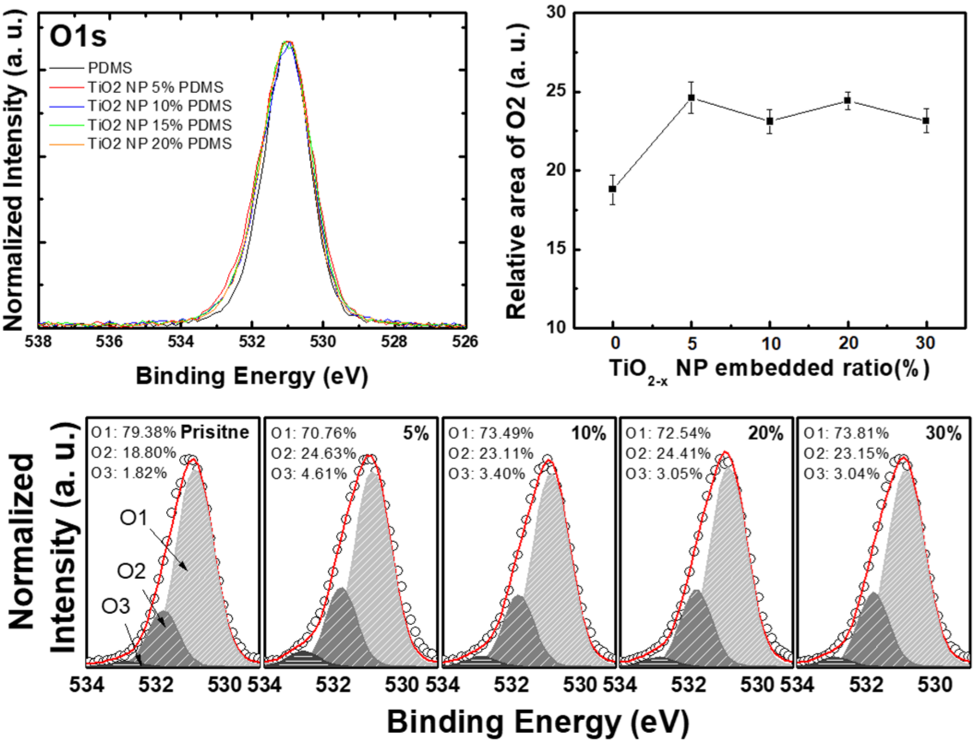

Figure S3. Change of oxygen deficient states with various TiO_{2-x} NP concentrations.

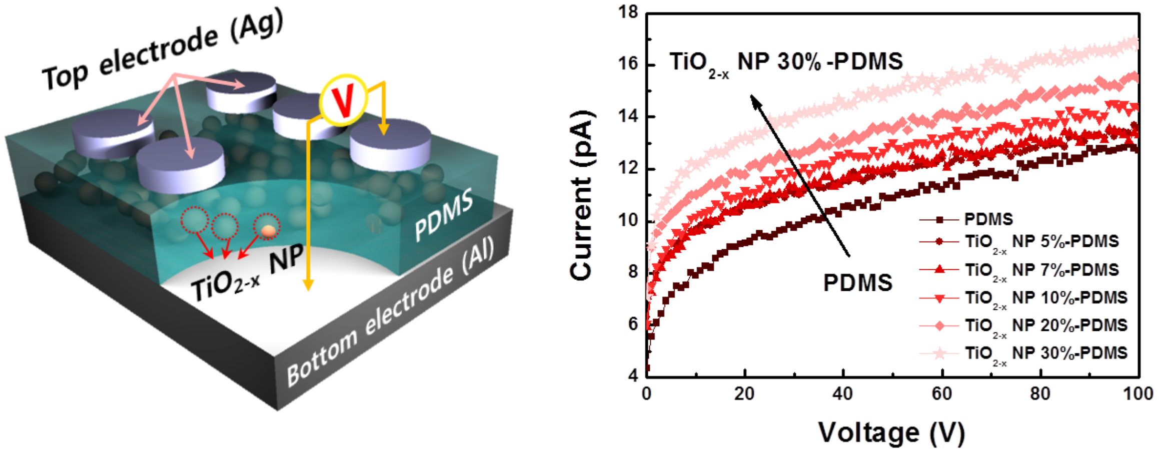

Figure S4. Leakage current characteristics of PDMS layer as a function of TiO_{2-x} NPs embedded ratio.
